# A Curricular Bioinformatics Approach to Teaching Undergraduates to Analyze Metagenomic Datasets Using R

**DOI:** 10.3389/fmicb.2020.578600

**Published:** 2020-09-10

**Authors:** Anne E. Kruchten

**Affiliations:** Department of Biology, The College of St. Scholastica, Duluth, MN, United States

**Keywords:** bioinformatics, curriculum, undergraduate, R, big data, metagenomics, biology

## Abstract

Biologists with bioinformatic skills will be better prepared for the job market, but relatively few biology programs require bioinformatics courses. Inclusion in the curriculum may be hindered by several barriers, including lack of faculty expertise, student resistance to computational work, and few examples in the pedagogical literature. An 8-week wet-lab and *in silico* research experience for undergraduates was implemented. Students performed DNA purification and metagenomics analysis to compare the diversity and abundance of microbes in two samples. Students sampled snow from sites in northern Minnesota and purified genomic DNA from the microbes, followed by metagenomic analysis. Students used an existing metagenomic dataset to practice analysis skills, including comparing the use of Excel versus R for analysis and visualization of a large dataset. Upon receipt of the snow data, students applied their recently acquired skills to their new dataset and reported their results via a poster. Several outcomes were achieved as a result of this module. First, YouTube videos demonstrating hands-on metagenomics and R techniques were used as professional development for faculty, leading to broadened research capabilities and comfort with bioinformatics. Second, students were introduced to computational skills in a manner that was intentional, with time for both introduction *and* reinforcement of skills. Finally, the module was effectively included in a biology curriculum because it could function as either a stand-alone course or a module within another course such as microbiology. This module, developed with Course-based Undergraduate Research Experience guidelines in mind, introduces students and faculty to bioinformatics in biology research.

## Introduction

In 1920, botanist Hans Winkler coined the term “genome” as a fusion of the words gene and chromosome (Winkler, 1920). Since that time, the “omics” fields have exploded, creating such terms as “pseudome” (the population of pseudogenes), “translatome” (the population of proteins in the cell, weighted by their abundance level), and many others that are increasingly becoming a normal part of the lexicon for biologists^1^. The term “bioinformatics” was defined by Luscombe et al. (2001), as “conceptualizing biology in terms of molecules (in the sense of Physical chemistry) and applying “informatics techniques” (derived from disciplines such as applied maths, computer science and statistics) to understand and organize the information associated with these molecules, on a large scale. In short, bioinformatics is a management information system for molecular biology and has many practical applications.” Undergraduates in biology should be trained in this field to successfully compete in the job market and make vital contributions to the biological sciences as their careers mature.

The *Vision and Change: A Call to Action* report of 2011 (Brewer and Smith, 2011) emphasized that undergraduate biology students should have competence in computational and systems level approaches and the ability to use large databases. Only a small fraction of institutions offer a full undergraduate bioinformatics program (Mellon, 2020), but several offer courses on bioinformatics. In the state of Minnesota, 50% of public and private school biology departments offer a bioinformatics course in their curriculum but none appear to require it for the degree. This may reflect a lack of expertise among faculty to teach the course.

In 2016-17, 116,759 bachelor’s degrees in biology were conferred to graduates in the United States (US-DOE, 2020). Among bachelor’s degree holders 25–29 years old, biology graduates’ annual salaries were not significantly different than the median annual income of all degree holders of $50,600, but computer and information science degree holders had an annual income of $70,100, well above the median income (NCES, 2020). A slight increase in biology-related computer information jobs is predicted, suggesting that biology majors would be well-served to develop computer information skills to complement their biology degrees (Araneo et al., 2017).

Bioinformatics is a broad field that encompasses gene alignment tools, crowdsourcing approaches, metagenomics, and many others. Rather than lecturing about bioinformatics, many groups have chosen to incorporate bioinformatics tools into CUREs (Course-based Undergraduate Research Experiences). In CUREs, students are working in classes on research projects of interest to the broader scientific community (Auchincloss et al., 2014). On the CUREnet website^2^, several bioinformatics CUREs have been shared for faculty adoption and participation, including a CRISPR-Cas9 project^3^, a study of iron uptake in insects^4^, Genome Solver: Microbial Comparative Genomics^5^, and the Genomics Education Partnership (GEP)^6^. These programs, and many others across the country, teach students a variety of gene-based bioinformatics approaches including using BLAST, multiple gene alignment, primer design, and many others. Students develop strong gene analysis skills while also contributing to active scientific research projects in the process.

While these CUREs develop students’ genome analysis skills, other courses focus on microbiome analyses including mapping microbiomes of the human oral cavity (Wang et al., 2015) and crowdsourcing datasets of antibiotic resistance in microbes (Freeman et al., 2016; Small-World, 2020). Students in these courses develop research skills such as bacterial culturing, sterile technique, PCR, and hypothesis building. Few projects, however, teach undergraduates the computational skills required to statistically analyze “big data” in biological fields.

Computational skills are required to analyze and find patterns in big data, which includes the four Vs: volume of data, velocity of processing the data, variability of data sources, and veracity of the data quality. Applications of big data analysis can be found everywhere, but for biologists especially important applications include genome sequencing, ecological studies (such as of microbiomes), and health care information (Li and Chen, 2014). Graduates of biology programs have opportunities for employment in any of these fields but may not have the important computational skills in parallel with wet lab or field biology skills to be successful in big data fields. There seem to be few CUREs or similar programs published in the literature that provide instructions for how faculty can implement curricular modules to help students develop these big data skills.

Several groups have outlined a series of bioinformatics competencies for life scientists, including CourseSource (the Bioinformatics Learning Framework) (Rosenwald et al., 2016), the Curriculum Task Force of the International Society of Computational Biology (ISCB) Education Committee (Mulder et al., 2018), and the Network for Integrating Bioinformatics into Life Sciences Education (NIBLSE) (Wilson Sayres et al., 2018). Building on previous work from both CourseSource and ISCB, NIBLSE surveyed instructors at US institutions and used the data to develop a list of Core Competencies for Undergraduate Life Scientists. While many of the core competencies focus on genomics-based bioinformatics skills, several of the competencies are addressed by the work in this project. The competencies are listed below (Wilson Sayres et al., 2018), and the bolded ones are addressed by the approach in this project:

• **C1. Explain the role of computation and data mining in addressing hypothesis-driven and hypothesis-generating questions within the life sciences.**
• C2. Summarize key computational concepts, such as algorithms and relational databases, and their applications in the life sciences.
• C3. Apply statistical concepts used in bioinformatics.
• C4. Use bioinformatics tools to examine complex biological problems in evolution, information flow, and other important areas of biology.
• C5. Find, retrieve, and organize various types of biological data.
• **C6. Explore and/or model biological interactions, networks, and data integration using bioinformatics.**
• **C7. Use command-line bioinformatics tools and write simple computer scripts.**
• C8. Describe and manage biological data types, structure, and reproducibility.
• C9. Interpret the ethical, legal, medical, and social implications of biological data.

Importantly, both the CourseSource Bioinformatics Learning Framework and the ISCB Curriculum Task Force recognize that there are different levels of users of bioinformatics curriculum, including bioinformatics *engineers*, bioinformatics *scientists*, and bioinformatics *users*. The approach described here is geared toward bioinformatics *users*, including both faculty who are interested in learning about these tools and students who will be moving forward into a variety of careers in research, medicine, education, and others. This course module is a starting point for introducing students to low level Bloom’s taxonomy areas such as knowledge and comprehension of bioinformatics. It is hoped that this introduction will spark an interest in students to learn more about the field and become bioinformatics scientists. This approach is also intended to provide an entry point for faculty to begin developing new courses in bioinformatics within their undergraduate biology programs and collaborate with colleagues in computer science fields to pool interests and resources.

## Bioinformatics Course Module

In response to the need for a big data CURE, I have developed an 8 week course that meets for two 2-hour sessions weekly in which students gain hands-on experience using R and Excel to analyze large datasets. To mimic an authentic research experience as closely as possible, the 10 students work as a research group as they discuss the literature, develop hypotheses, and plan experiments. Individuals or pairs are responsible for collecting samples and performing the actual sample preparation and experiments. Data analysis is completed individually and then discussed and improved in the full research group. While this course was developed as a stand-alone experience, it could easily be incorporated as a module in a broader full length course.

The primary student learning outcome for this course was to develop students’ data science skills using Excel and R. The premise of this research course was to perform a metagenomic analysis of the microbiota in two different snow samples. To accomplish this research project, students perform a literature review, develop hypotheses, collect and prepare samples, perform metagenomic sequencing (through a third party vendor), learn data analysis skills, and present their research findings via a poster presentation. Secondary student learning outcomes for this course include those described in the CURE network: making discoveries of interest to the broader scientific community, an iterative work experience, communication of their findings, and development of scientific research skills (CUREnet, 2020).

### Weeks 1 and 2: Literature Review, Hypothesis Development, and Sampling

Table 1 highlights the main activities completed in the course, beginning with a literature review. Because the primary learning outcome for this course is the development of R and Excel skills, the instructor can assist in the literature review process by developing the initial research question and providing some preliminary resources to begin the discussion. In this project, I developed the initial research question of “how does the bacterial population vary between two snow samples from different locations on campus?” and provided several primary and secondary articles about microbiomes, microorganisms often found in snow, and bacterial abundance and diversity. Students used these resources as jumping off points to find more sources (usually PDFs, websites, and videos) which were collected in a class Google folder. Students visually mapped these sources into three broad categories on the whiteboard: “snow,” “microbiomes,” and “microbial diversity.” After a group discussion, each student was responsible for developing an individual literature review from these and other sources they found.

**TABLE 1:** Flow of the bioinformatics course.

Week #	Course topics
1	Literature review
	*The literature review is initiated by the instructor to save time and is further developed by students.*
2	Hypothesis development, sampling, and sample preparation
	*Students use their literature review to develop their hypothesis, identify sampling methods, and prepare DNA samples, allowing a pairing of wet lab skills with in silico activities.*
	*When wet lab resources are unavailable, this step can be completed by the instructor or replaced with an existing publicly available dataset.*
3	Understanding metagenomic sequencing and big data
	*Students build on foundational knowledge of DNA from prerequisite courses by viewing video material on PCR and sequencing.*
4	Introduction of Data Analysis Skills
	*Instruction in statistics, Excel, and R using a combination of video material and in class discussions builds a foundation of data analysis skills.*
5	Practice data analysis using an existing dataset
	*Students use their developing data analysis skills to mimic the instructors’ actions using Excel and R to analyze an existing dataset.*
6	Reinforcing data analysis skills with snow dataset
	*Students apply the data analysis skills they have learned and practiced to a new dataset from samples collected on campus.*
7	Creating a Scientific Poster of Analysis of Big Data
	*Students showcase all the skills practiced in the course in a poster containing a research question, background material, a hypothesis, methods, results, and discussion.*
8	Poster presentations
	*Students complete the semester by recording a video presentation of themselves presenting their poster. If available, students also present their posters at a campus-wide research symposium.*

This fast-paced literature review process leads to the development of a research question, hypothesis, and sampling procedure. Metagenomic analysis with our vendor takes 3–4 weeks, so it was essential to collect and prepare samples right away to allow time for the primary student learning outcome of developing skills in Excel and R. To this end, after discussion, most of the class agreed upon the same research question and hypothesis, with slight variations that could be accommodated within the sampling and sample preparation processes. Our research question asked if the microbiota of snow samples would differ between an area heavily trafficked by both foot and automobile traffic compared to campus trails primarily traveled by snowshoe. Most students hypothesized that the area with both foot and automobile traffic would have more bacteria overall and more diversity of bacteria. Students demonstrated their understanding of the field and our research question development by submitting a draft of an introduction for their final poster project (see Supplementary Material Section 5 for teaching materials).

Sampling and sample purification were relatively simple and inexpensive. Students used 50 ml plastic conical tubes (VWR 89039-656) to collect three samples spaced at one meter intervals along a line at each of the two sites for 3 days in a row. To purify microbial DNA from the samples, the snow was melted and filtered through a 0.2 micron polyester membrane using an Aeropress coffee press^7^. The membranes containing the filtered microorganisms were then processed using the Qiagen DNeasy PowerWater kit (Qiagen 14900-50-NF). After confirming the presence of bacterial DNA via PCR with a 16S primer set (idtdna.com; 16S rRNA For #51-01-19-06, 16S rRNA Rev 51-01-19-07), the samples were sent for metagenomic sequencing off campus.

### Weeks 3 and 4: Introducing Metagenomics, Big Data, and R

The first step in teaching students about bioinformatics was to guide them through an understanding of how metagenomic sequencing works and how the dataset was generated. A prerequisite for this course was a one semester Foundations in Biology course covering the essentials of molecular biology, including central dogma concepts such as DNA, RNA, base pairing, replication, and transcription. The Supplementary Material contain a list of resources used for reviewing foundational DNA and PCR knowledge (Supplementary Material Section 1). With this background in mind, students work to understand the polymerase chain reaction, or PCR. This foundational knowledge is essential, in part because it strips away the complexities of how we typically teach replication with emphases on all of the different enzymes (polymerases I and III, primase, ligase, helicase, etc.) and focuses on the simple concept of creating a complement sequence of DNA to the template.

After mastering PCR, students then move on to understanding DNA sequencing, beginning with Sanger sequencing. To do this, students watch a series of YouTube videos on Sanger Sequencing^8^, the evolution of next-generation sequencing^9^, and finally Illumina sequencing^10^ used by our vendor (see Supplementary Material Section 1 for more details). After watching the video on Illumina sequencing, students usually express a combination of fascination and confusion. To provide further practice in understanding this extremely important process, we break into student pairs and have each pair illustrate the processes of cluster formation on whiteboards using color coding. After performing a similar exercise to better understand base calling, we complete this section of the instruction by discussing how multiple overlapping DNA segments from one organism can be used to generate the sequence for the entire 16S ribosomal RNA gene.

It is common for biology students in our program to have a fear or aversion to mathematical and other quantitative or computational approaches. 65% of traditional undergraduate students enrolled in our college identify as female, 31% identify as first generation college students, 35% have family incomes less than $50,000, and 70% come from rural communities and small cities. Many students have taken the minimum mathematics courses required by the state graduation guidelines. In a study of life sciences majors conducted by Andrews and Aikens (Andrews and Aikens, 2018), both females and first generation students exhibited a lower interest in mathematics topics in biology than their counterparts, and females perceived a higher cost associated with doing math in biology than their male counterparts. They also found that students’ likelihood of taking a biostatistics class was positively related to their interest and perceived utility of the course. A goal for this course module is to spark future interest in bioinformatics training, so it was important to demonstrate to students the utility of statistical analysis both for the project and their future careers.

In recognition of these factors, I began the bioinformatics instruction with a review (or novel instruction) of basic statistical analysis. To accomplish this, students first reviewed major statistical functions such as mean, median, standard deviation, standard error, *p*-values, and Student’s *t*-test using a freely available resource compiled by MIT^11^. These concepts were practiced using a very simple assignment completed in pairs during class time examining the statistical significance of simple drug treatment data (see Supplementary Material Section 2 for details). In class discussion helped to sort out problems in understanding before moving on to larger dataset analysis.

Next, students are introduced to fundamental concepts in data analysis, including data clean up and developing the research question. To facilitate this process, I provided the students with a dataset previously collected in the Boundary Waters Canoe Area Wilderness (BWCAW). This dataset included triplicate sampling of four different sample sites resulting in 12 columns of data on a spreadsheet. After metagenomic sequencing, 15,000 unique bacterial species or OTUs (operational taxonomic units) were identified in the spreadsheet rows, resulting in 180,000 unique cells of data. Given that most students’ experience of using Excel to this point had been in traditional lab courses, this was by far the largest Excel file any of them had ever opened.

To make the experience less overwhelming for the students, I provide them with a version of the dataset that condensed OTUs into phyla, resulting in a dataset with 12 sampling columns and 23 rows of identified phyla. My goal was for them to be able to use Excel to average the triplicate results from each sample site and make comparisons across the data, either between the four individual sample sites or between phyla. To do this, I created a video of myself using Excel to average the sites, perform a *t*-test comparing the data between sites, and then sort the data by increasing *p*-value, thus reordering the data so that the most significant *p*-values were at the top of the list. Students then were required to repeat the actions of this video on both the phyla dataset and the OTU dataset. In doing this, students gained experience cleaning up and renaming columns, writing formulas, accessing the formula bank, sorting, and visualizing data.

### Weeks 5 and 6: Practicing and Reinforcing Big Data Analysis Skills

After establishing comfort with analyzing data in Excel, we moved on to R. R is a freely available statistical computing program (The R-Foundation, 2020) used across many fields for the analysis and visualization of data. For the purposes of this course module, I wanted to introduce students to the pros and cons of using the programming language R versus using Excel both for data analysis and for data visualization, particularly for its ability to generate a heat map of large datasets. This includes establishing student knowledge, but not necessarily application, of using a command line and understanding the function of packages, bundles of shareable code created by experts in the field and freely available for use.

When students learned coding was involved, there was an immediate sense of anxiety in the room. To alleviate this stress, I returned to an approach with which the students were familiar: learning by watching videos. Just as they had learned to use Excel functions by watching me perform tasks via video, the basics of R were laid out by watching a series of publicly available YouTube videos. Many videos are available, but I chose the “R Programming for Beginners” playlist from the R Programming 101 YouTube channel^12^ (see the Supplementary Material Section 3 for a complete list of videos). In this series, the host, public health specialist Greg Martin, guides viewers through the whole process of using R, including downloading R and R Studio onto their computers, learning basic commands such as identifying variables and manipulating a preloaded dataset of health characteristics of Star Wars characters, and installing and using R packages. This playlist resonated with the students, both because of the clear instructions and because of the link to public health, a field with which many of the biology students could identify. Students watched this series of videos on their own and their sole assignment was to replicate exactly what the host did and turn in a screenshot of their final R Studio product.

**FIGURE 1:**
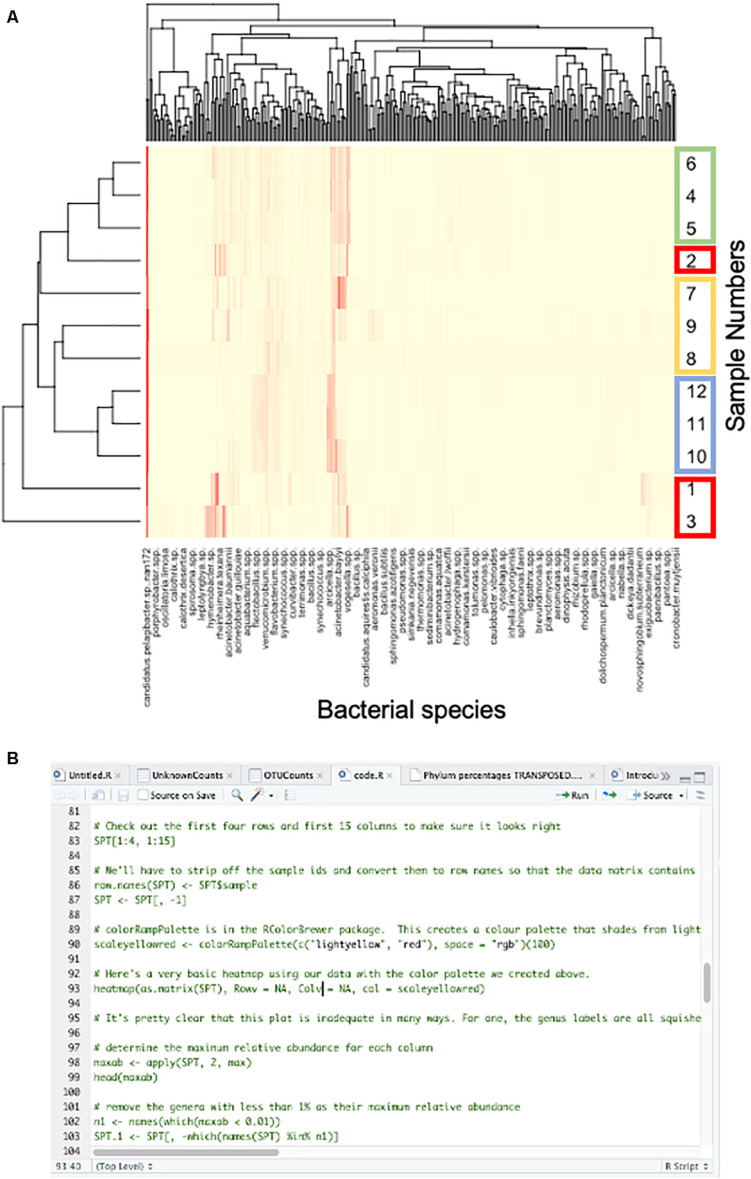
Example heat map and R code. Students used R to generate two heat maps in the course, first with a practice set of data from the Boundary Waters Canoe Area Wilderness which was followed by a heat map of snow sample data to reinforce skills. **(A)** Representative student-generated heat map of the BWCAW data. On the right axis, triplicate samples are boxed with corresponding colors; bacterial species’ names are on the bottom axis. R-generated dendrograms are on the left and top axes. **(B)** A snapshot of the script window of R studio showing the code students used to generate the heat maps. A full copy of the code is available in Supplementary Material.

Once the students achieved some initial comfort with R, I gave them a fully composed sheet of code to copy and paste into the script window of R. The code was created by modifying freely available code (Albert and Yoder, 2013), including the packages gplots, vegan, and RColorBrewer to plot data, create the heatmap, and apply a color scheme. I used this approach for three reasons. First, students did not yet have the capability to compose their own code because they didn’t have enough knowledge of syntax to do what was needed. Second, because R is an open access community, students and instructors can find existing code for many functions on the internet and modify it to fit their needs. Third, by providing code that was annotated (with # lines explaining each line of code), students were able to walk through each line of code, understand the function, and run the code to achieve a final product of a heat map demonstrating the diversity and abundance of microbial samples across sampling sites in the BWCAW (Figure 1; full code in the Supplementary Material Section 4). Because the purpose of this course module was to introduce bioinformatics users to command line coding, the ability to generate a finished product was important both to increase their level of confidence in using R and in order to demonstrate the analysis capabilities available in R that were not available in Excel.

At this point in the course, students had participated in a strong introduction to data analysis using both Excel and R. They had manipulated a dataset larger than any of them had seen before and reflected on the pros and cons of each tool in analyzing the datasets. Each student had observed Excel and R being used via video and followed up with practice completing the work themselves. This iterative approach follows best practices in pedagogy where students are offered multiple opportunities to observe, practice, and learn a skill.

When the data from the metagenomic analysis of the snow samples was returned to us in week six, students were ready to analyze it. The final project was a standard scientific poster presentation of their background, research question, hypothesis, methods, results, and discussion. To accomplish this task, students had to return to the notes they took for the analysis of the BWCAW dataset and apply these approaches for the snow dataset. This task involved cleaning up the data, and properly labeling sample columns, and changing existing lines of code in R to import the proper.csv file, identifying columns correctly and creating an appropriate visualization. By using this iterative approach of first observing, then practicing, and finally applying, all the students were able to successfully assign the right syntax to the code and create a successful project.

### Potential for Virtual Course Delivery

As presented, this process allows students to experience both wet bench and *in silico* research. However, it is important to note that the project could be modified to include only the *in silico* experience for students, as was the case in the second iteration of this course in spring 2020 due to the COVID-19 pandemic and the closure of college facilities. It would be possible to provide this experience with the many publicly available datasets, but during the college closure I chose to perform the wet bench portion myself prior to the beginning of the course so that students felt they had a more “personal” sample rather than a dataset to which they had no personal attachment. This approach resonated with students as evidenced in their comments in the course evaluations.

During the COVID-19 pandemic in Spring 2020, the course was delivered using both asynchronous and synchronous (Zoom) methods. The course meeting schedule was altered to limit Zoom fatigue by meeting synchronously on Tuesdays and working asynchronously on course materials during the remainder of the week. Thursday meeting sessions were reserved for open office hours, an approach that well was received by students and widely used. Tuesday synchronous meetings were initially used for discussions of the overall project, research design, and sequencing videos. Breakout rooms in Zoom were used extensively to facilitate small group discussions of research questions and to build comprehension of the sequencing videos. Beginning in week 3, the course took on essentially a “flipped” format. Students viewed and practiced skills introduced in the videos and synchronous class time was used for troubleshooting, comprehension checks, and setting up the “next steps.” Students in this virtual course were still able to successfully use R for statistical analysis and visualization of their data.

## Discussion

One of the most important take home messages of this work is that we should take advantage of technology both to continue skill development as faculty and to teach resourcefulness to students. Many faculty who teach undergraduate students completed their dissertations before the age of bioinformatics or in an area that did not focus on quantitative skills. These faculty may not currently possess the skills to incorporate a bioinformatics module into a course. YouTube affords faculty the opportunity to learn new skills in a step-by-step manner when the technology and approaches may be wholly new to them. This is a very inexpensive and efficient way to acquire professional development that can serve to enhance both classroom teaching and potential new areas of research.

As part of the course evaluation, students were asked to answer a series of confidence questions about skills developed in the course (Table 2). On a scale of 1–10, with 10 being high confidence, all students rated themselves as a ten when asked about confidence in pipetting a variety of liquids with micropipettors, reflecting the skills developed in the wet lab portion of the course. When asked about explaining Sanger sequencing and Next Generation Sequencing to another scientist, the class averages were 6.8 and 6.7 out of 10, respectively, for these new skills learned in the course. The course successfully introduced students to basic knowledge about R, as reflected in an 8.9 average score to “I can copy, perform, and run a simple code in R.” As expected from an 8 week introductory module, the students did not feel confident enough to create and run their own R code (average score 5.2).

**TABLE 2:** Student confidence in course skills.

How confident are you that you can complete the following tasks?	Class average confidence Scale of 1–10; 10 = high confidence
I can pipet a variety of liquids with micropipetors.	10
I can understand scientific methods and instructions to perform experiments.	9.4
I could explain the process of PCR to another scientist.	8.7
I can explain Sanger sequencing to another scientist.	6.8
I can explain next generation sequencing (NGS) to another scientist.	6.7
I can analyze the bands on a gel to determine if I got the right sized DNA product from PCR.	9.5
I can use Excel to perform a *t*-test and analyze a *p*-value.	8.4
I can pose a question and use a large data set to effectively answer that question.	8.3
I can copy, perform, and run a simple code in R.	8.9
I can create and run my own code in R.	5.2

Students were also asked, “After completing this course, how has your interest in biological research changed?” All of their free response answers are below:

• I am still interested in it, and now realize the importance of being able to effectively use R and excel to convey my data.
• My interest in research has stayed quite high after taking this course. I am planning on working in the more biochemical side, but this was still very interesting and helped me make sure that a career in research is where I belong.
• I feel like I have a better understanding of how questions are being asked in the biological community.
• My interest in biological research has grown even stronger. I knew before that I love research, but every time I continue to do it, my passions grow stronger.
• It greatly raised my interest in biological research. It was cool to see how the experiments we performed gave us numbers, that we could find relationships between.
• I was always curious about how scientists made the figures they did. After using R, examining larger datasets is a lot less frightening.
• I have a greater understanding of the importance of microbiomes and am interested in my own microbiome!
• I was very hesitant about research before this course because I had a few bad experiences, but this class changed my outlook on it. I am definitely more interested and would like to do more.

• My interest has greatly increased in biological research, specifically, on human microbiomes like the gut microbiota. Also, conducting my own biological research and experiencing the challenges of creating a poster has made me appreciate all the hard work scientist do to give us informative papers.
• I am once again excited now about the medical applications of molecular biology and studies! I’m excited to skim new articles and have a better toolbox to understand them after learning about R and how microbiome data can be represented.

While this course had a very small sample size (*n* = 10), these responses suggest that this approach to using R was positively received by students. Moreover, the students saw a utility in learning R, which research shows may lead to continued interest in participating in mathematical biology experiences (Andrews and Aikens, 2018).

In a short time frame, the course introduced students to bioinformatics and provided an opportunity for further practice. Because of the students’ ability to effectively visualize the dataset with R, they were able to think critically about the data and consider future research questions. From the R-generated heat map, the students realized that their initial hypothesis was incorrect. The heavy foot and automobile traffic sample site did have a higher abundance of bacteria but the diversity of bacteria was much lower than the sample site with light traffic. Several students continued their analysis of the data even after the course ended and proposed a new research question for the next offering of the course.

Several outcomes were achieved as a result of this module. First, faculty expertise was enhanced in a time efficient manner using YouTube training videos, leading to broadened research capabilities and comfort. Second, students were introduced to computational skills in a manner that was effective and intentional, with time for both introduction *and* reinforcement of skills. Finally, the module was effectively included in a biology curriculum because it could function as either a stand-alone course or a module within another course such as microbiology, leading to flexibility in the curriculum. This module, developed with CURE guidelines in mind, is an effective and easily implementable way to introduce a broad group of students to bioinformatics in biology research, and also serves as a springboard for interested students to pursue further training and research in bioinformatics.

## Data Availability Statement

The original contributions presented in the study are included in the article/Supplementary Material, further inquiries can be directed to the corresponding author.

## Author Contributions

The author confirms being the sole contributor of this work and has approved it for publication.

## Conflict of Interest

The author declares that the research was conducted in the absence of any commercial or financial relationships that could be construed as a potential conflict of interest.

## Footnotes

1 http://bioinfo.mbb.yale.edu/what-is-it/omes/

2 https://serc.carleton.edu/curenet/whatis.html

3 https://serc.carleton.edu/curenet/collection/213026.html

4 https://serc.carleton.edu/curenet/institutes/boulder/examples/207018.html

5 https://serc.carleton.edu/curenet/collection/218072.html

6 https://serc.carleton.edu/curenet/collection/215335.html

7 https://aeropress.com

8 https://www.youtube.com/watch?v=Jnk_4Maf5Fk

9 https://www.youtube.com/watch?v=jFCD8Q6qSTM&t=176s

10 https://www.youtube.com/watch?v=fCd6B5HRaZ8

11 https://web.mit.edu/∼csvoss/Public/usabo/stats_handout.pdf

12 https://www.youtube.com/channel/UCfJyQ3P2k_SuqfxVdqIEQNw
