# Supplementary material for: A Curricular Bioinformatics Approach to Teaching Undergraduates to Analyze Metagenomic Datasets Using R
Source: Front Microbiol. 2020 Sep 10;11:578600. doi: 10.3389/fmicb.2020.578600 (PMC7511545; doi:10.3389/fmicb.2020.578600)
Supplement: Supplementary file 1 [file Data_Sheet_1.PDF]

R-code for creating a heatmap from metagenomic analysis data

If you're new to R, there's great news! Because R is part of an open access community, you can search the internet and find existing code for many different functions. Search "R code for heatmap" and you'll find many websites that include not only code but tutorials on the functions. You can then modify the code to fit your needs.

In the code below, any line that begins with # is not an actual code line but rather a descriptor. You can use these to provide instructions for students.

Basic heatmap code modified from various sources.

```
install.packages("gplots")
library(gplots)
source("http://bioconductor.org/biocLite.R")
biocLite("Heatplus"")
library(Heatplus)
library(vegan)
library(RColorBrewer)


PhylumPercentsTransposed <- read.csv("Phylum percentages TRANSPOSED.csv")
head(PhylumPercentsTransposed)
dim(PhylumPercentsTransposed)

PhylumPercentsTransposed[1:3, 1:4]
row.names(PhylumPercentsTransposed) <- PhylumPercentsTransposed$sample
PhylumPercentsTransposed <- PhylumPercentsTransposed[, -1]
dim(PhylumPercentsTransposed)
PhylumPercentsTransposed[1:3, 1:4]

data.prop <- PhylumPercentsTransposed/rowSums(PhylumPercentsTransposed)
data.prop[1:3, 1:3]

PhylumPercentsTransposed[1:3, 1:4]

scaleyellowred <- colorRampPalette(c("lightyellow", "red"), space = "rgb")(100)

heatmap(as.matrix(data.prop), Rowv = NA, Colv = NA, col = scaleyellowred)

heatmap(as.matrix(data.prop), Rowv = NA, Colv = NA, col = scaleyellowred, margins =
c(10, 2))

data.dist <- vegdist(data.prop, method = "bray")
```

```
library(vegan)
install.packages("vegan")
library(vegan)
data.dist <- vegdist(data.prop, method = "bray")

row.clus <- hclust(data.dist, "aver")

heatmap(as.matrix(data.prop), Rowv = as.dendrogram(row.clus), Colv = NA, col =
scaleyellowred, margins = c(10, 3))

data.dist.g <- vegdist(t(data.prop), method = "bray")
col.clus <- hclust(data.dist.g, "aver")
heatmap(as.matrix(data.prop), Rowv = as.dendrogram(row.clus), Colv =
as.dendrogram(col.clus), col = scaleyellowred, margins = c(10, 3))

install.packages("gplots")
install.packages("Bioconductor")
library(gplots)

# to install packages from Bioconductor:
source("http://bioconductor.org/biocLite.R")
biocLite("Heatplus")  # annHeatmap or annHeatmap2
library(Heatplus)

# load the vegan package for hierachical clustering if you want to use distance functions
not specified in dist.
library(vegan)

# load the RColorBrewer package for better colour options
library(RColorBrewer)

# load our data Species Percentages Transposed and call it SPT -- remember it the .csv
file must be in your working folder first!
SPT <- read.csv("Species percentages TRANSPOSED.csv")

# look at the dimensions of the data (number of rows vs columns) using the dim
command
dim(SPT)
# ours read out [1] 12 1237, meaning twelve rows and 1237 columns

# Check out the first four rows and first 15 columns to make sure it looks right
SPT[1:4, 1:15]

# We'll have to strip off the sample ids and convert them to row names so that the data
matrix contains only sequence count data.
row.names(SPT) <- SPT$sample
```

```
SPT <- SPT[, -1]

# colorRampPalette is in the RColorBrewer package.  This creates a colour palette that shades from light yellow to red in RGB space with 100 unique colours
scaleyellowred <- colorRampPalette(c("lightyellow", "red"), space = "rgb")(100)

# Here's a very basic heatmap using our data with the color palette we created above.
heatmap(as.matrix(SPT), Rowv = NA, Colv = NA, col = scaleyellowred)

# It's pretty clear that this plot is inadequate in many ways. For one, the genus labels are all squished along the bottom and impossible to read. One solution to this problem is to remove genera that are exceedingly rare from this figure. Let's try removing genera whose relative read abundance is less than 1% of at least 1 sample.

# determine the maximum relative abundance for each column
maxab <- apply(SPT, 2, max)
head(maxab)

# remove the genera with less than 1% as their maximum relative abundance
n1 <- names(which(maxab < 0.01))
SPT.1 <- SPT[, -which(names(SPT) %in% n1)]

# the margins command sets the width of the white space around the plot. The first element is the bottom margin and the second is the right margin
heatmap(as.matrix(SPT.1), Rowv = NA, Colv = NA, col = scaleyellowred, margins = c(10, 2))

# This is better, but there's still many different species names.  You can check how many with the dim command as in the beginning of the code.
dim(SPT.1)
# Our result is 12 rows, 789 columns.  Let's see what happens if we make the cutoff 2%.

# determine the maximum relative abundance for each column
maxab <- apply(SPT, 2, max)
head(maxab)

# remove the genera with less than 2% as their maximum relative abundance
n1 <- names(which(maxab < 0.02))
SPT.2 <- SPT[, -which(names(SPT) %in% n1)]

# the margins command sets the width of the white space around the plot. The first element is the bottom margin and the second is the right margin
heatmap(as.matrix(SPT.2), Rowv = NA, Colv = NA, col = scaleyellowred, margins = c(10, 2))

dim(SPT.2)
```

```
# down to 569 columns. Let's try a 4% cutoff.

# determine the maximum relative abundance for each column
maxab <- apply(SPT, 2, max)
head(maxab)

# remove the genera with less than 4% as their maximum relative abundance
n1 <- names(which(maxab < 0.04))
SPT.4 <- SPT[, -which(names(SPT) %in% n1)]

# the margins command sets the width of the white space around the plot. The first
element is the bottom margin and the second is the right margin
heatmap(as.matrix(SPT.4), Rowv = NA, Colv = NA, col = scaleyellowred, margins =
c(10, 2))
dim(SPT.4)
# 427 columns!  Let's go big and try a 8% cutoff!

# determine the maximum relative abundance for each column
maxab <- apply(SPT, 2, max)
head(maxab)

# remove the genera with less than 8% as their maximum relative abundance
n1 <- names(which(maxab < 0.08))
SPT.8 <- SPT[, -which(names(SPT) %in% n1)]

# the margins command sets the width of the white space around the plot. The first
element is the bottom margin and the second is the right margin
heatmap(as.matrix(SPT.8), Rowv = NA, Colv = NA, col = scaleyellowred, margins =
c(10, 2))
dim(SPT.8)
# 291 columns.  I'm good with that for now.


# Now let’s add a dendrogram for the samples. The heatmap function will do this for
you, but I prefer to make my own using the vegan package as it has more options for
distance metrics. Also, this means that you can do hierarchical clustering using the full
dataset, but only display the more abundant taxa in the heatmap.
install.packages("vegan")
library(vegan)

# calculate the Bray-Curtis dissimilarity matrix on the full dataset:
SPT.dist <- vegdist(SPT, method = "bray")

# Do average linkage hierarchical clustering. Other options are 'complete' or 'single'.
You'll need to choose the one that best fits the needs of your situation and your data.
row.clus <- hclust(SPT.dist, "aver")
```

```
# make the heatmap with Rowv = as.dendrogram(row.clus)
heatmap(as.matrix(SPT.8), Rowv = as.dendrogram(row.clus), Colv = NA, col =
scaleyellowred, margins = c(10, 3))

# You can also add a column dendrogram to cluster the genera that occur more often
together. Note that this one must be done on the same dataset that is used in the
Heatmap (i.e. reduced number of genera).

# you have to transpose the dataset to get the genera as rows
SPT.dist.g <- vegdist(t(SPT.8), method = "bray")
col.clus <- hclust(SPT.dist.g, "aver")

# make the heatmap with Rowv = as.dendrogram(row.clus)
heatmap(as.matrix(SPT.8), Rowv = as.dendrogram(row.clus), Colv =
as.dendrogram(col.clus), col = scaleyellowred, margins = c(10, 3))
```
