# Supplementary material for: A Curricular Bioinformatics Approach to Teaching Undergraduates to Analyze Metagenomic Datasets Using R
Source: Front Microbiol. 2020 Sep 10;11:578600. doi: 10.3389/fmicb.2020.578600 (PMC7511545; doi:10.3389/fmicb.2020.578600)
Supplement: Supplementary file 2 [file Data_Sheet_2.PDF]

Supplementary Materials

Table of Contents

1. Resources for teaching PCR, Sequencing, and Illumina Sequencing
2. Resources for teaching about statistics
3. Video playlist for teaching R
4. R-code for creating a heatmap from metagenomic analysis data
5. Instructions for the Poster Project and Peer Review

1. Resources for teaching PCR, Sequencing, and Illumina Sequencing

Using a Micropipet
Bio-Rad Laboratories
https://www.youtube.com/watch?v=p-OPOYbeZP0

What is Molecular Biology? Wikipedia page of definitions and general knowledge
https://en.wikipedia.org/wiki/Molecular_biology

DNA and RNA - Part 1
Bozeman Science
https://www.youtube.com/watch?v=qoERVSWKmGk

DNA and RNA - Part 2
Bozeman Science
https://www.youtube.com/watch?v=W4mYwsr9gGE

Our World in Data
Global Change Data Lab
https://ourworldindata.org/coronavirus?fbclid=IwAR1PR5AGLsEFn1ybQsPZJx5ApXlj65w9dLNDQn2pIjXwMU_pzMF5hFn2riM

DNA Isolation - Simple Animated Tutorial
Mr. Simple Science
https://www.youtube.com/watch?v=8cYvyYOjzOc

The Kavli Foundation – the Microbiome
https://www.kavlifoundation.org/microbiome

microBEnet: the microbiology of the Built Environment network
https://microbe.net/

Sequencing and the American Gut Project | Illumina MiSeq, 16S rRNA
https://www.youtube.com/watch?v=1uZtCMY-yEw

PCR (Polymerase Chain Reaction) Tutorial - An Introduction
Applied Biological Materials
https://www.youtube.com/watch?v=matsiHSuoOw

DNA sequencing | Biomolecules | MCAT | Khan Academy
Khanacademymedicine
https://www.youtube.com/watch?v=Jnk_4Maf5Fk

Next Generation Sequencing (NGS) - An Introduction
Applied Biological Materials
https://www.youtube.com/watch?v=jFCD8Q6qSTM&t=176s

Illumina Sequencing by Synthesis
Illumina
https://www.youtube.com/watch?v=fCd6B5HRaZ8

2. Resources for teaching about statistics

Statistics Cheat Sheet from MIT
https://web.mit.edu/~csvoss/Public/usabo/stats_handout.pdf

Activity: Using Excel to Practice TTEST skills

For each of the data sets below (which are identical in all three problems), consider the scenario presented. Think about what the possible distribution curves might look like. Does the scenario vary the curve? Does the scenario vary the tails or the pairing? Use the Statistics Cheat Sheet from MIT for additional information.

For each data set, run a Ttest and compare your answers, using $p<0.05$ as statistically significant.

For assistance, use the formula builder which provides info on tails and pairing. Look on the "formula" tab for help.

How does Drug A vs Drug B effect the concentration of Bad Protein X in the blood?
Numbers are ug/ml of Bad Protein X in the blood after treatment; each row is a different patient.

Drug A	Drug B
38	32
52	39
48	40
25	47
39	41
51	34
46	30
55	36
46	36
53	38
45	46
42	58
54	52
65	29
56	40
67	46
40	46
32	45
43	40
34	40
45	49

How effective is Drug A vs Drug B in reducing Bad Protein X in the blood?
Numbers are ug/ml of Bad Protein X in the blood after treatment; each row is a different patient.

Drug A	Drug B
38	32
52	39
48	40
25	47
39	41

51	34
46	30
55	36
46	36
53	38
45	46
42	58
54	52
65	29
56	40
67	46
40	46
32	45
43	40
34	40
45	49

How does Drug A affect Bad Protein X levels if given to the same patient before and after a meal?
Numbers are ug/ml of Bad Protein X in the blood after treatment; each row is a different patient.

Drug A before meal	Drug A after a meal
38	32
52	39
48	40
25	47
39	41
51	34
46	30
55	36
46	36
53	38
45	46
42	58
54	52
65	29
56	40
67	46
40	46
32	45
43	40
34	40
45	49

3. Video playlist for teaching R

All videos are from the "R programming for beginners" series by Greg Martin found on the R Programming 101 channel (https://www.youtube.com/c/RProgramming101/channels)

Teaching Tip: It's important that students work through these videos in order of the playlist. Each one builds on the previous video. The author introduces a dataset of Star Wars characters that is included within one of the packages.

These videos provide step by step instructions for both faculty and students to become comfortable with R.

Playlist:

R programming for beginners – Why you should use R
https://www.youtube.com/watch?v=9kYUGMg_14s&list=PLtL57Fdbwb_Chn-dNR0qBjH3esKS2MXY3&index=2&t=81s

How to install R and install R Studio. How to use R studio | R programming for beginners
https://www.youtube.com/watch?v=orjLGFmx6l4&list=PLtL57Fdbwb_Chn-dNR0qBjH3esKS2MXY3&index=2

How to import data and install packages. R programming for beginners.
https://www.youtube.com/watch?v=e8B9YU_M5FM&list=PLtL57Fdbwb_Chn-dNR0qBjH3esKS2MXY3&index=3

How to import data from excel into R studio. R programming for beginners
https://www.youtube.com/watch?v=cnD1op2Oo3M&list=PLtL57Fdbwb_Chn-dNR0qBjH3esKS2MXY3&index=4

R programming for beginners. Manipulate data using the tidyverse: select, filter and mutate
https://www.youtube.com/watch?v=nRtp7wSEtJA&list=PLtL57Fdbwb_Chn-dNR0qBjH3esKS2MXY3&index=5

Data types in R programming
https://www.youtube.com/watch?v=VtUVQWl0aRA&list=PLtL57Fdbwb_Chn-dNR0qBjH3esKS2MXY3&index=6

R programming for beginners: Rename variables and reorder columns. Data cleaning and manipulation
https://www.youtube.com/watch?v=q_ax_aG6ZdQ&list=PLtL57Fdbwb_Chn-dNR0qBjH3esKS2MXY3&index=7

Recoding data using R programming. Using the tidyverse and dplyr packages to create a new variable
https://www.youtube.com/watch?v=KQuPsYHG1TI&list=PLtL57Fdbwb_Chn-dNR0qBjH3esKS2MXY3&index=8

10 data filtering tips using R programming. Use the tidyverse to filter and subset your data.
https://www.youtube.com/watch?v=pU10ghMvAuM&list=PLtL57Fdbwb_Chn-dNR0qBjH3esKS2MXY3&index=9

4. R-code for creating a heatmap from metagenomic analysis data

If you're new to R, there's great news! Because R is part of an open access community, you can search the internet and find existing code for many different functions. Search "R code for heatmap" and you'll find many websites that include not only code but tutorials on the functions. You can then modify the code to fit your needs.

In the code below, any line that begins with # is not an actual code line but rather a descriptor. You can use these to provide instructions for students.

Packages and palettes:
For more information on the packages used in this code, please use the following links:
Vegan: https://cran.r-project.org/web/packages/vegan/vegan.pdf
Gplots: https://cran.r-project.org/web/packages/gplots/gplots.pdf
RColorBrewer: https://www.rdocumentation.org/packages/RColorBrewer/versions/1.1-2/topics/RColorBrewer

Basic heatmap code modified from various sources.

```
install.packages("gplots")
library(gplots)
source("http://bioconductor.org/biocLite.R")
biocLite("Heatplus"")
library(Heatplus)
library(vegan)
library(RColorBrewer)


PhylumPercentsTransposed <- read.csv("Phylum percentages TRANSPOSED.csv")
head(PhylumPercentsTransposed)
dim(PhylumPercentsTransposed)

PhylumPercentsTransposed[1:3, 1:4]
row.names(PhylumPercentsTransposed) <- PhylumPercentsTransposed$sample
PhylumPercentsTransposed <- PhylumPercentsTransposed[, -1]
dim(PhylumPercentsTransposed)
PhylumPercentsTransposed[1:3, 1:4]

data.prop <- PhylumPercentsTransposed/rowSums(PhylumPercentsTransposed)
data.prop[1:3, 1:3]

PhylumPercentsTransposed[1:3, 1:4]

scaleyellowred <- colorRampPalette(c("lightyellow", "red"), space = "rgb")(100)

heatmap(as.matrix(data.prop), Rowv = NA, Colv = NA, col = scaleyellowred)

heatmap(as.matrix(data.prop), Rowv = NA, Colv = NA, col = scaleyellowred, margins = c(10, 2))

data.dist <- vegdist(data.prop, method = "bray")

library(vegan)
```

```
install.packages("vegan")
library(vegan)
data.dist <- vegdist(data.prop, method = "bray")

row.clus <- hclust(data.dist, "aver")

heatmap(as.matrix(data.prop), Rowv = as.dendrogram(row.clus), Colv = NA, col = scaleyellowred, margins =
c(10, 3))

data.dist.g <- vegdist(t(data.prop), method = "bray")
col.clus <- hclust(data.dist.g, "aver")
heatmap(as.matrix(data.prop), Rowv = as.dendrogram(row.clus), Colv = as.dendrogram(col.clus), col =
scaleyellowred, margins = c(10, 3))

install.packages("gplots")
install.packages("Bioconductor")
library(gplots)

# to install packages from Bioconductor:
source("http://bioconductor.org/biocLite.R")
biocLite("Heatplus")  # annHeatmap or annHeatmap2
library(Heatplus)

# load the vegan package for hierachical clustering if you want to use distance functions not specified in dist.
library(vegan)

# load the RColorBrewer package for better colour options
library(RColorBrewer)

# load our data Species Percentages Transposed and call it SPT -- remember it the .csv file must be in your
working folder first!
SPT <- read.csv("Species percentages TRANSPOSED.csv")

# look at the dimensions of the data (number of rows vs columns) using the dim command
dim(SPT)
# ours read out [1] 12 1237, meaning twelve rows and 1237 columns

# Check out the first four rows and first 15 columns to make sure it looks right
SPT[1:4, 1:15]

# We'll have to strip off the sample ids and convert them to row names so that the data matrix contains only
sequence count data.
row.names(SPT) <- SPT$sample
SPT <- SPT[, -1]

# colorRampPalette is in the RColorBrewer package.  This creates a colour palette that shades from light yellow
to red in RGB space with 100 unique colours
scaleyellowred <- colorRampPalette(c("lightyellow", "red"), space = "rgb")(100)

# Here's a very basic heatmap using our data with the color palette we created above.
heatmap(as.matrix(SPT), Rowv = NA, Colv = NA, col = scaleyellowred)
```

It's pretty clear that this plot is inadequate in many ways. For one, the genus labels are all squished along the bottom and impossible to read. One solution to this problem is to remove genera that are exceedingly rare from this figure. Let's try removing genera whose relative read abundance is less than 1% of at least 1 sample.

```
# determine the maximum relative abundance for each column
maxab <- apply(SPT, 2, max)
head(maxab)

# remove the genera with less than 1% as their maximum relative abundance
n1 <- names(which(maxab < 0.01))
SPT.1 <- SPT[, -which(names(SPT) %in% n1)]

# the margins command sets the width of the white space around the plot. The first element is the bottom margin and the second is the right margin
heatmap(as.matrix(SPT.1), Rowv = NA, Colv = NA, col = scaleyellowred, margins = c(10, 2))

# This is better, but there's still many different species names.  You can check how many with the dim command as in the beginning of the code.
dim(SPT.1)
# Our result is 12 rows, 789 columns.  Let's see what happens if we make the cutoff 2%.

# determine the maximum relative abundance for each column
maxab <- apply(SPT, 2, max)
head(maxab)

# remove the genera with less than 2% as their maximum relative abundance
n1 <- names(which(maxab < 0.02))
SPT.2 <- SPT[, -which(names(SPT) %in% n1)]

# the margins command sets the width of the white space around the plot. The first element is the bottom margin and the second is the right margin
heatmap(as.matrix(SPT.2), Rowv = NA, Colv = NA, col = scaleyellowred, margins = c(10, 2))

dim(SPT.2)
# down to 569 columns. Let's try a 4% cutoff.

# determine the maximum relative abundance for each column
maxab <- apply(SPT, 2, max)
head(maxab)

# remove the genera with less than 4% as their maximum relative abundance
n1 <- names(which(maxab < 0.04))
SPT.4 <- SPT[, -which(names(SPT) %in% n1)]

# the margins command sets the width of the white space around the plot. The first element is the bottom margin and the second is the right margin
heatmap(as.matrix(SPT.4), Rowv = NA, Colv = NA, col = scaleyellowred, margins = c(10, 2))
dim(SPT.4)
# 427 columns!  Let's go big and try a 8% cutoff!
```

```
# determine the maximum relative abundance for each column
maxab <- apply(SPT, 2, max)
head(maxab)

# remove the genera with less than 8% as their maximum relative abundance
n1 <- names(which(maxab < 0.08))
SPT.8 <- SPT[, -which(names(SPT) %in% n1)]

# the margins command sets the width of the white space around the plot. The first element is the bottom margin and the second is the right margin
heatmap(as.matrix(SPT.8), Rowv = NA, Colv = NA, col = scaleyellowred, margins = c(10, 2))
dim(SPT.8)
# 291 columns.  I'm good with that for now.


# Now let’s add a dendrogram for the samples. The heatmap function will do this for you, but I prefer to make my own using the vegan package as it has more options for distance metrics. Also, this means that you can do hierarchical clustering using the full dataset, but only display the more abundant taxa in the heatmap.
install.packages("vegan")
library(vegan)

# calculate the Bray-Curtis dissimilarity matrix on the full dataset:
SPT.dist <- vegdist(SPT, method = "bray")

# Do average linkage hierarchical clustering. Other options are 'complete' or 'single'. You'll need to choose the one that best fits the needs of your situation and your data.
row.clus <- hclust(SPT.dist, "aver")

# make the heatmap with Rowv = as.dendrogram(row.clus)
heatmap(as.matrix(SPT.8), Rowv = as.dendrogram(row.clus), Colv = NA, col = scaleyellowred, margins = c(10, 3))

# You can also add a column dendrogram to cluster the genera that occur more often together. Note that this one must be done on the same dataset that is used in the Heatmap (i.e. reduced number of genera).

# you have to transpose the dataset to get the genera as rows
SPT.dist.g <- vegdist(t(SPT.8), method = "bray")
col.clus <- hclust(SPT.dist.g, "aver")

# make the heatmap with Rowv = as.dendrogram(row.clus)
heatmap(as.matrix(SPT.8), Rowv = as.dendrogram(row.clus), Colv = as.dendrogram(col.clus), col = scaleyellowred, margins = c(10, 3))
```

5. Instructions for the Poster Project and Peer Review

Introduction Instructions

The purpose of a poster's introduction is to help the viewer understand big concepts in the field of study and how your particular poster is going to provide more information.

For this assignment, please include:

- Four to five bullet points that describe the field as a whole, and
- One bullet point that highlights your hypothesis
- An image that informs the viewer about the field of study. Examples could include an image depicting snowflake formation, weather patterns, etc. The image should not be too broad or off topic, for example a picture of a snowflake or storm cloud.

To describe the field in your bullet points consider the following questions:

1. What is the microbiome?
2. Where is the microbiome found?
3. Why is the microbiome important?
4. How do we study the microbiome?
5. What is the research question for our study?
6. Why is our research question important?

References should be cited using any format with which you are comfortable. Include both in text references (common styles include a superscript or (name date)) and a list of corresponding references in any style format (CSE, APA, etc) as long as they're a consistent style throughout the poster.

I highly encourage creating this assignment in Powerpoint or similar software. This will make it easy to transfer to your poster later on. Font on posters should be at minimum 18 point for ease of reading, if not greater. You're welcome to use more than one slide.

Please submit this assignment via Blackboard by 10 am on Tuesday, March 31st.

Elements of the Introduction: Peer Review

How well are each of the following components addressed? Please rate each category from Poor to Excellent and provide constructive feedback.

7. What is the microbiome?

   Not present Poor Satisfactory Very Good Excellent

8. Where is the microbiome found?

   Not present Poor Satisfactory Very Good Excellent

9. Why is the microbiome important?

Not present Poor Satisfactory Very Good Excellent

10. How do we study the microbiome?

Not present Poor Satisfactory Very Good Excellent

11. What is the research question for our study?

Not present Poor Satisfactory Very Good Excellent

12. Why is our research question important?

Not present Poor Satisfactory Very Good Excellent

13. How well does the selected image summarize this project?

Not present Poor Satisfactory Very Good Excellent

Methods section instructions

This methods section is for a poster, so it should be a *graphical* representation of the methods, NOT a fully textual representation. Create a flow chart of methods images that demonstrates how we moved from sample collection to sequencing.

Include in your methods section:

- Hypothesis
- Sample collection
- DNA purification
- PCR reaction
- Gel analysis
- Metagenomic sequencing

Use resources from Blackboard for the details and names of the protocols.

Using images from the internet is perfectly appropriate as long as you properly cite them.

Text should be incorporated to facilitate understanding.

Your final poster will be 2'x3' in size. The methods will likely take up about 1/3 of the whole poster. Create your methods section in a powerpoint file with a slide size of 2'x3'; change the slide size using the custom setting under page set up.

Bring one printed copy of your methods section on a 8.5x11" sheet of paper to class on Thursday 4/11 and upload one copy to Blackboard.

Methods: Peer Review

How well are each of the following components addressed? Please rate each category from Poor to Excellent and provide constructive feedback.

14. Is the hypothesis clearly stated? Is it a testable hypothesis?

Not present Poor Satisfactory Very Good Excellent

15. Is it clear how and where samples were collected?

Not present Poor Satisfactory Very Good Excellent

16. Are the steps of DNA purification identified? Is it clear how DNA is purified from other cell components?

Not present Poor Satisfactory Very Good Excellent

17. Is it clear that PCR was performed specifically using 16S rRNA primers?

Not present Poor Satisfactory Very Good Excellent

18. Is it clear that we're looking for a 16S band on the gel?

Not present Poor Satisfactory Very Good Excellent

19. Is the metagenomics sequencing adequately displayed?

Not present Poor Satisfactory Very Good Excellent

20. How well does the methods section flow from one step to the next?

Not present	Poor	Satisfactory	Very Good	Excellent

21. Is there sufficient text to guide the reader but not so much as to take away from the images?

Not present	Poor	Satisfactory	Very Good	Excellent

22. Are images useful for the story being told and are they cited?

Not present	Poor	Satisfactory	Very Good	Excellent

Final Poster Project

The purpose of a scientific poster is to convey information about your ongoing project including your background, your question and hypothesis, your approach, and your results and conclusion. When we as scientists present our research, it is important to engage our audience through good visuals, well written text, and an enthusiastic presentation. Your final poster project will provide you an opportunity to do all these things.

Required Elements of the Poster

- Title
- Name
- Question/Hypothesis
- Introduction
- Methods
- Results – include graphics and your calculations/approach
- Discussion/Conclusion
- References

Poster size = 24” x 36”, vertical or horizontal
Poster submission via PDF to Blackboard by noon on Thursday, May 9th.
Poster should be aesthetically pleasing and free of errors.

If you choose to use Excel for your analysis:

1. Ask two separate but related questions
2. Include two analyses to answer your two questions
3. At least one of your questions must use the species data set
4. Represent your data in graphical form
5. Include your approach (for example, did you narrow the data with a t-test first, by sorting, etc.)

If you choose to use R for your analysis:

1. Ask one question
2. Include one analysis to answer your question
3. Represent your data in graphical form
4. Include your approach (for example, did you narrow the data with a t-test first, by sorting, etc.)
5. Include your code

Poster presentation

Record yourself presenting your poster.

- You can choose to be in the presentation visually or just include your voice.
- Minimum time: 3 minutes
- Maximum time: 5 minutes
- Describe each part of your project completely, keeping in mind that your audience is a scientifically literate group of your peers.

Options for recording your presentation:
Zoom
Screencastomatic
Many others….
